# Automated image analysis reveals the dynamic 3-dimensional organization of multi-ciliary arrays

**DOI:** 10.1242/bio.014951

**Published:** 2015-12-23

**Authors:** Domenico F. Galati, David S. Abuin, Gabriel A. Tauber, Andrew T. Pham, Chad G. Pearson

**Affiliations:** Department of Cell and Developmental Biology, University of Colorado School of Medicine, 2801 East 17th Ave, Aurora, CO 80045-2537, USA

**Keywords:** *Tetrahymena*, Basal body, Centriole, Cilia, Polarity, Poc1, Automated image analysis

## Abstract

Multi-ciliated cells (MCCs) use polarized fields of undulating cilia (ciliary array) to produce fluid flow that is essential for many biological processes. Cilia are positioned by microtubule scaffolds called basal bodies (BBs) that are arranged within a spatially complex 3-dimensional geometry (3D). Here, we develop a robust and automated computational image analysis routine to quantify 3D BB organization in the ciliate, *Tetrahymena thermophila*. Using this routine, we generate the first morphologically constrained 3D reconstructions of *Tetrahymena* cells and elucidate rules that govern the kinetics of MCC organization. We demonstrate the interplay between BB duplication and cell size expansion through the cell cycle. In mutant cells, we identify a potential BB surveillance mechanism that balances large gaps in BB spacing by increasing the frequency of closely spaced BBs in other regions of the cell. Finally, by taking advantage of a mutant predisposed to BB disorganization, we locate the spatial domains that are most prone to disorganization by environmental stimuli. Collectively, our analyses reveal the importance of quantitative image analysis to understand the principles that guide the 3D organization of MCCs.

## Abstract (precis)

**Summary:** We develop an automated computational image analysis routine to quantify basal body organization, which detects subtle spatial phenotypes resulting from environmental and genetic perturbations.

## INTRODUCTION

Multi-ciliary arrays are collections of tens to thousands of motile cilia that generate hydrodynamic force (Marshall and Kintner, 2008). These arrays of cilia clear mucus from the respiratory tract, propel cerebrospinal fluid through brain ventricles and establish the leftward flow of embryonic fluid (Nonaka et al., 1998; Sawamoto et al., 2006; Tilley et al., 2015). Cilia are nucleated and stabilized by microtubule scaffolds known as basal bodies (BBs). BBs comprise nine rotationally symmetric triple microtubules that form a cylinder approximately 200 nm in diameter and 500 nm in length (Bettencourt-Dias et al., 2011). During cilium formation, a BB docks at the plasma membrane, acquires polarized auxiliary structures and nucleates the cilium's axoneme (Ishikawa and Marshall, 2011). BBs organize multi-ciliary arrays by specifying where cilia form and by dictating the direction of ciliary beating.

Cilia and BBs must be assembled correctly for multi-ciliary arrays to develop the appropriate density and spatial organization of cilia (Marshall and Kintner, 2008; Park et al., 2008; Pearson, 2014). For example, deletion of the conserved BB component Centrin 2 disrupts ependymal cilia organization leading to decreased hydrodynamic flow in brain ventricles (Ying et al., 2014). Similarly, depletion of CCDC78, which mediates *de novo* BB assembly in *Xenopus* epithelial cells, also reduces cilia density and multi-cilia generated flow (Klos Dehring et al., 2013; Zhao et al., 2013). Following assembly, BBs are stabilized to withstand the forces generated by ciliary beating. The need for BB stabilization has been demonstrated by a loss of cilia when steady-state multi-ciliary arrays are depleted of BB stability factors such as Poc1 or Bld10/CEP135 (Bayless et al., 2012; Pearson et al., 2009b). The BB's rotational orientation defines the direction of ciliary beating, and to produce a strong net flow, BBs must be aligned along a common axis (Chien et al., 2013; Marshall and Kintner, 2008; Mitchell et al., 2007). Bbof1, a factor that localizes to polarized structures adjacent to BBs, is both necessary and sufficient for aligning BBs with cellular polarity (Chien et al., 2013), while DisA1p, a component of BB-associated striated fibers, promotes striated fiber elongation and resists BB disorientation (Galati et al., 2014). The importance of BB alignment is underscored by orientation defects found in patients with primary cilia dyskinesia that disrupt mucus clearance without impacting cilia beat frequency (Rayner et al., 1996). Proper BB assembly and orientation is critical for generating hydrodynamic force, but the comprehensive analysis of BB organization is difficult due to the large number of BBs that must be identified and quantified.

*Tetrahymena thermophila* is a unicellular ciliate that employs multi-ciliary arrays for propulsion in an aqueous environment (Frankel, 2000; Hennessey and Lampert, 2012). A single *Tetrahymena* cell contains approximately 550-650 BBs depending on the stage of cell division (Nanney, 1971; Nanney and Chow, 1974). 150 BBs are tightly packed into a feeding structure called the oral apparatus that defines the cell's anterior end (Frankel, 1964; Orias and Pollock, 1975), while 400-500 cortical BBs are aligned into 18-21 longitudinal ciliary rows that span the length of the cell (Frankel, 2008; Nanney, 1975; Pearson and Winey, 2009). BBs duplicate during cell division to produce two cells with a fully formed oral apparatus and an equal complement of cortical BBs (Nanney, 1971). Progression through the cell cycle can be monitored by the structure of the newly developing oral apparatus, which begins as a so called anarchic field of BBs and develops into a functional oral apparatus by the time of cell division [Stages I-IV, (Williams and Scherbaum, 1959); Stages I-VI, (Frankel, 1964)]. New cortical BBs (daughters) arise from the anterior surface of pre-existing mother BBs (Allen, 1969; Dippell, 1967; Nanney, 1975; Perlman, 1973). As they mature, daughter BBs separate from their mother in the anterior direction to occupy the next position within a ciliary row, in a process that is guided by inherent BB asymmetries (Pearson, 2014). Single daughter BBs are not produced at each mother in a 1:1 ratio. Rather, mother BBs within the medial regions of the cell give rise to multiple daughter BBs, which leads to an unequal distribution of mother and daughter BBs with more daughter BBs distributed to the posterior daughter cell (Kaczanowski, 1978; Nanney, 1971, 1975; Perlman, 1973). Although detailed characterizations of *Tetrahymena* BB morphogenesis have been performed, virtually all have sampled BBs in a defined cellular spatial domain and then extrapolated to other parts of the cell (Nanney, 1971; Nanney and Chow, 1974; Nanney et al., 1978). Few, if any, studies have attempted to analyze all the BBs of *Tetrahymena* cells.

**Abbreviations**

3D: 3-dimensional
BB: basal body
MIP: maximum intensity projection

Here, we quantify BB organization to understand how genetic and environmental factors impact the morphology of multi-ciliary arrays. However, manual analysis of BBs is time-consuming and requires significant human input. Here, we report an automated image analysis routine that quantifies the spatial distribution of *Tetrahymena* cortical BBs and reconstructs *Tetrahymena* 3D cellular geometry. We use this approach to generate novel insights into how the spatial organization of multi-ciliary arrays changes throughout the cell cycle and in response to genetic and environmental perturbations.

## RESULTS

### Identifying *Tetrahymena* BBs and their organization

**Fig. 1.**
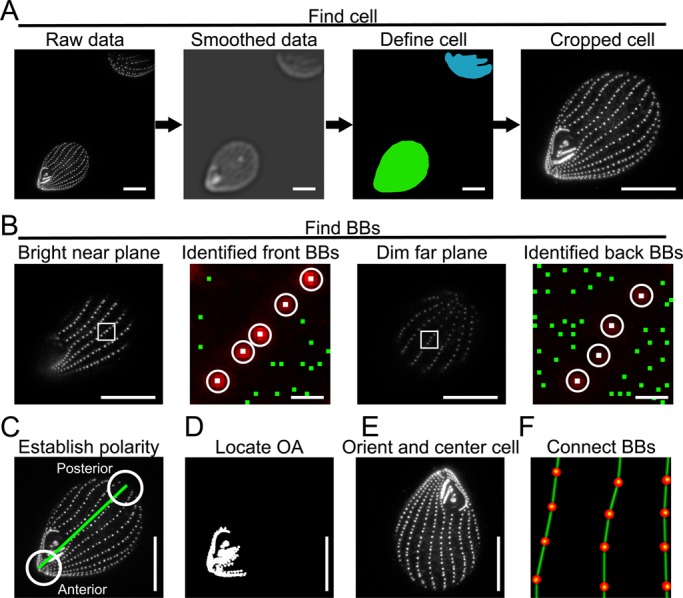
**An image analysis routine to reconstruct *Tetrahymena* multi-ciliary arrays.** (A) *Tetrahymena* are identified from raw images (left) by blurring the BB marker centrin with a large radius smoothing kernel (middle left) and identifying objects whose shape match stereotypical *Tetrahymena* (green cell, middle right). A bounding box of the cell outline is enlarged and used to crop the image stack (right). Scale bar: 15 µm. (B) Individual BBs are identified on both sides of the cell despite a significant decrease in fluorescence intensity on the back half of the cell. Grey scale images are single planes from the near and far halves of the cell displayed using the same intensity range. Color images are the enlarged boxed regions to show the scaled centrin signal (red), individual local maxima (green) and the maxima that the analysis routine identified as BBs (white circles). Scale bar: 15 µm or 1 µm (inset). (C) The anteroposterior axis is defined by the BB pairs that exhibit the greatest separation distance (green line). Anterior and posterior poles are determined by integrating the intensity within a circle centered over each of the poles and assigning the circle with the greatest intensity to be the anterior pole. Scale bar: 15 µm. (D) The oral apparatus is identified by its closely spaced BBs which appear as a continuous cluster of signal-containing voxels in a thresholded binary image. Scale bar: 15 µm. (E) The cell's polarity is used to rotate the cell so it is oriented along its anteroposterior axis. Scale bar: 15 µm. (F) Individual BBs (red spheres) are connected to their most logical anterior partner (green lines) using geometric constraints.

We developed a computational image processing routine to quantify the 3D organization of BBs in *Tetrahymena* multi-ciliary arrays by automating laborious tasks that typically require significant user input. *Tetrahymena* cells are identified and extracted from digital image stacks that are contaminated by partial cells and cellular debris (Fig. 1A). BBs are identified using an adaptive threshold calculated from the intensity profile of all maxima within a local neighborhood (Fig. 1B, Fig. S1). This approach reduces the errors caused by using a single intensity threshold for thick (>15 µm) *z*-stacks that suffer from signal attenuation due to light scattering as the *z*-plane moves further from the coverslip (Hell et al., 1993; Waters, 2009). Next, the cell's longitudinal axis is determined by identifying the BB pairs that exhibit the greatest separation distance from the cell anterior to posterior (Fig. 1C). Cellular polarity is then defined using the intensity profile of the anterior pole's closely spaced BBs (Fig. 1D, Fig. S2A,B). Finally, cellular polarity cues and simple geometry are used to connect each BB to its anterior and posterior partners within each ciliary row of the multi-ciliary array (Fig. 1E,F). The image analysis routine is robust as demonstrated by its ability to function on both wide-field and confocal images (Fig. S2C,D). Moreover, the routine identifies BBs and establishes connections with appropriate neighboring BBs at a level comparable to a trained human observer (Fig. S3).

**Fig. 2.**
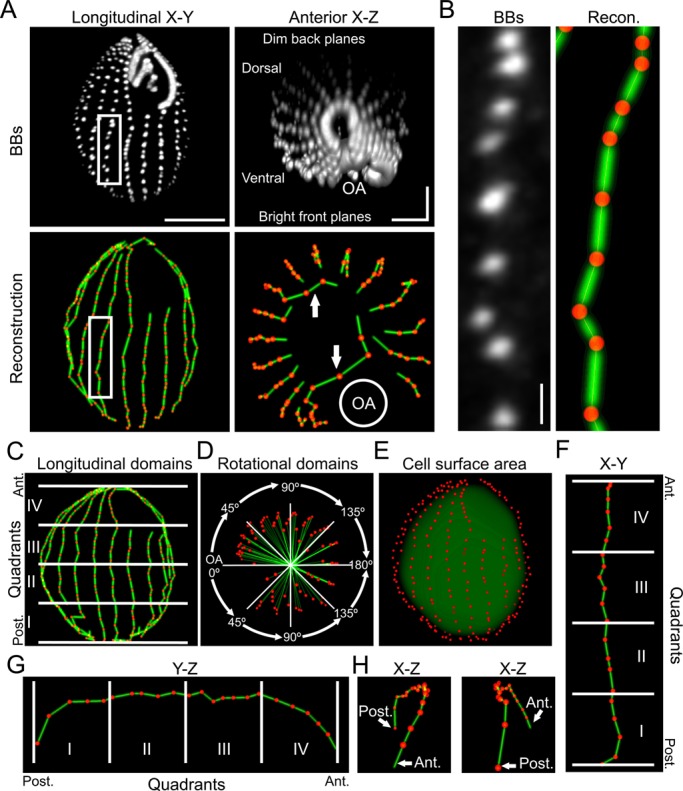
**Using *Tetrahymena* reconstructions to quantify multi-ciliary arrays.** (A) 3D renderings of a *Tetrahymena* cell generated from raw centrin data (grey images) and the corresponding reconstruction (red=BBs; green=connections between BBs). Renderings are shown in the longitudinal *x*-*y* dimension and the anterior *x*-*z* dimension to highlight the decreased fluorescence intensity on the back half of the cell. The reconstructed cell depicted in *x*-*y* and *x*-*z* shows the 3D model of the cell. BB connection failures (white arrows) are denoted at the anterior tip of the cell. OA, oral apparatus. Scale bar: 15 µm (*x*-*y*) or 5 µm (*x*-*z*) (B) Inset of the ciliary row highlighted in (A) and its corresponding reconstruction. Scale bar: 1 µm. (C) Each cell is divided into four quadrants (IV-I) along the anteroposterior axis and four rotational quadrants (0°, 45°, 90°, 135°) around the circumference of the cell oriented relative to the oral apparatus (OA) (D). (E) A 3D convex hull (green) is created from the BB coordinates (red) to estimate cellular surface area. The convex hull volume is uniformly decreased for visualization purposes. (F-H) An individual ciliary row is isolated and projected in the *x*-*y* (F), *y*-*z* (G) and *x*-*z* (H) dimensions to highlight the 3D tracing of individual BB connections across areas with cellular curvature.

The analysis routine produces a 3D reconstruction of the BBs comprising a *Tetrahymena* multi-ciliary array (Fig. 2A,B). These reconstructions are accurate on both the dorsal (side opposite the oral apparatus) and ventral (side containing the oral apparatus) surfaces despite the significant decrease in fluorescence intensity on the surface furthest from the coverslip (Fig. 2A). The one area where the reconstructions tend to be less accurate is the anterior pole where BB spacing between rows approaches that of BB spacing within a row (Fig. 2A, arrows). To quantify BB organization, we divided *Tetrahymena* cells into longitudinal and rotational spatial domains. The longitudinal domains are defined by the normalized linear distance along the anterior-posterior axis (Fig. 2C) (Kaczanowski, 1978; Pearson et al., 2009a) and the rotational domains are defined by angular rotation around the circumference of the cell relative to the oral apparatus (Fig. 2D). This allows each BB to be assigned to one of 16 unique spatial domains, facilitating the comparison of multiple cells. Furthermore, by calculating the 3D convex hull of the BBs, the routine approximates the cell surface area (Fig. 2E). Importantly, reconstructing BBs in 3D space preserves the curvature of the cell (Fig. 2F-H), which increases the accuracy of distance measurements compared to 2D projections or single planes, which has been problematic in prior analyses of *Tetrahymena* shape (Kaczanowski, 1978).

Long term imaging of *Tetrahymena* remains difficult because immobilization for extended periods of time has deleterious effects on cellular physiology, which hinders investigation of the assembly and dynamics of BBs. An alternative to live cell observations is analysis of staged fixed cells or organisms (Downs and Davies, 1993; Frankel, 1964; Williams and Scherbaum, 1959). During the cell cycle, *Tetrahymena* duplicate their BBs so that daughter cells receive a full complement of BBs after each cell division. Cortical BBs are added within ciliary rows while oral apparatus BBs are added to the medial region of the cell adjacent to the postoral ciliary row (Frankel, 1964; Kaczanowski, 1978). The development of the new oral apparatus can be used to group cells into four cell cycle stages (Stage I, II, III, and IV; Williams and Scherbaum, 1959). We took advantage of this and employed our analysis routine on staged wild-type and mutant cells to gain insight into how BB organization changes during the cell cycle and in different genetic mutants. We specifically focused on four aspects of BB organization: (1) density, (2) spacing, (3) alignment, and (4) fluorescent protein levels. Our results demonstrate the utility of automated image analysis to uncover subtle differences in the organization of multi-ciliary arrays.

### BB frequency during the cell cycle

**Fig. 3.**
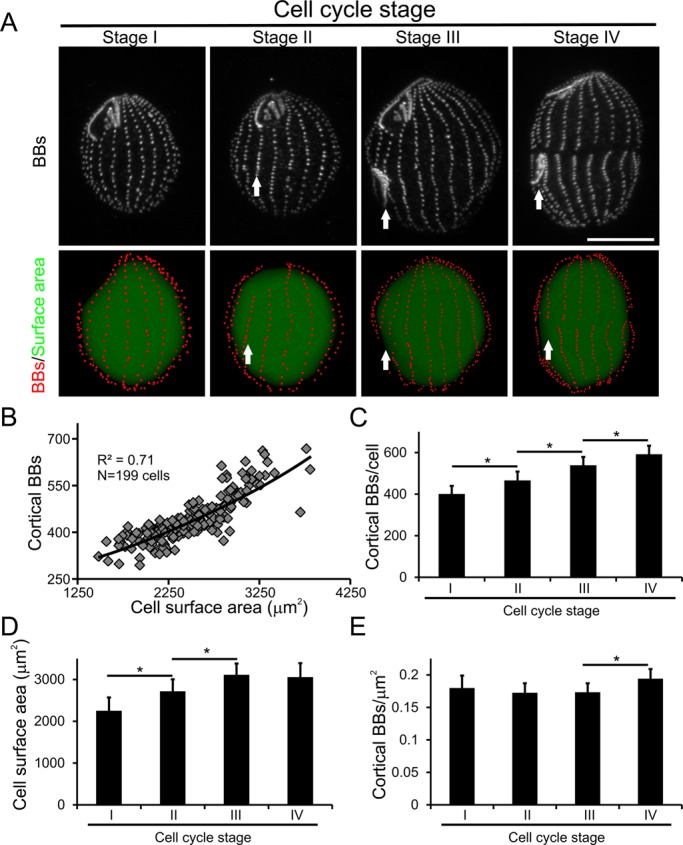
**Global analyses of cortical BBs reveals the relationship between BB addition and cell growth during the cell cycle.** (A) Cycling cells stained for centrin (top row) and cell cycle staged according to the development of the primordial oral apparatus. 3D reconstructions (bottom row) corresponding to each cell shows BBs (red) overlaid atop a shaded 3D convex hull (green) to show the cell surface area. White arrows denote the location of the primordial oral apparatus. Scale bar: 15 µm. (B) Scatter plot of cortical BBs plotted versus cell surface area reveals a linear relationship as the cell size increases. (C) The average number of cortical BBs per cell increases through each stage of cell division. (D) The cell surface area increased from Stages I to III and plateaus between Stages III and IV. (E) The increase in cortical BBs in Stage IV cells causes an increase in BB density due to the lack of surface area expansion. *N*=127 Stage I, *N*=42 Stage II, *N*=19 Stage III, *N*=11 Stage IV; these values represent the cumulative number of cells imaged from three separate cultures. *P<0.05, data is represented as mean±s.d. Significance between groups was determined with one-way ANOVA followed by a Tukey HSD post-test.

To understand how cortical BB frequency relates to changes in cell shape, we quantified the total number of cortical BBs in staged *Tetrahymena* cells (Fig. 3A). Prior analysis of whole-cell BB frequency achieved by manual sampling and extrapolation estimated that Stage I cells contain approximately 350-450 cortical BBs (Nanney et al., 1978). Our analysis produced similar values, with Stage I cells averaging 401 cortical BBs per cell (Fig. 3C). However, the analysis routine often misses the first two BBs of each ciliary row due to their close spacing (Fig. 2A). Since *Tetrahymena* contain roughly 20 ciliary rows, our BB counts underestimate the number of BBs by approximately 40. The average surface area quantified from the 3D convex hulls generated for Stage I cells is 2251±319 µm^2^ (Fig. 3D). This value is consistent with prior studies using a particle analyzer that found the *Tetrahymena* cell surface area to be approximately 1900 µm^2^ (Ricketts and Rappitt, 1974). We conclude that the 3D reconstructions produced by our image analysis routine are morphologically accurate compared to prior analyses achieved by manual quantification.

As *Tetrahymena* progress during the cell cycle they duplicate their cortical BBs and increase their size. However, the precise relationship between total cortical BB frequency and cell size is unknown. Regression analysis across all of the cell cycle stages revealed a strong linear relationship between the total number of cortical BBs and cell surface area (Fig. 3B, R^2^=0.71). We next measured the average surface area and cortical BB frequency for each cell cycle stage. The number of cortical BBs steadily increases from Stages I through IV (Fig. 3C), which is consistent with prior reports (Nanney, 1971). In contrast, surface area increases through Stage III before reaching a plateau by Stage IV (Fig. 3E). Therefore, cortical BB density remains constant, except for the last stage of cell division (Stage IV) when new BB assembly continues in the absence of surface area expansion.

### New BB assembly during the cell cycle

**Fig. 4.**
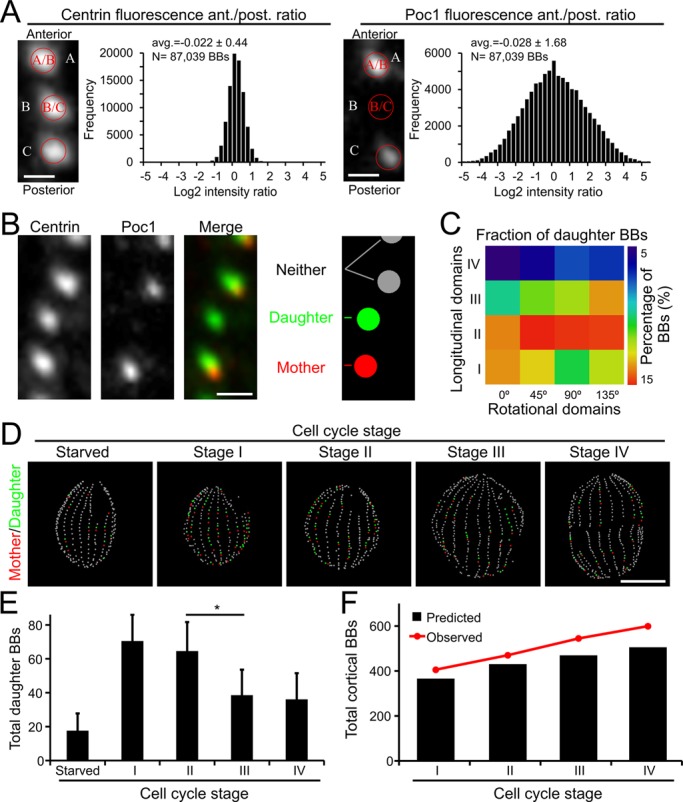
**The timing and spatial domains of new BB assembly.** (A) The image analysis routine measures the fluorescence intensity of fluorescently labeled BB components relative to the anteriorly positioned BB. Centrin (left) incorporation is rapid, which leads to a relatively uniform intensity ratio across all BB pairs. Poc1:GFP (right) incorporates in an age dependent profile, leading to greater distribution of intensity ratios and a broad intensity ratio distribution. Scale bar: 1 µm. *N* represents the total number of basal bodies from 199 cells imaged from 3 separate cultures. (B) To identify nascent daughter BBs, the analysis routine identifies BB pairs where Poc1 intensity in the anterior BB is 2 fold less than the posterior BB and assigns the posterior BB ‘mother’ status (red) and the anterior BB ‘daughter’ status (green). BBs that do not exhibit greater than a 2-fold Poc1 intensity differential are not assigned mother/daughter status (grey). Scale bar: 1 µm. (C) A spatial analysis of daughter BB position in cycling cells reveals the greatest enrichment in the medial and posterior quadrants. (D) Maps of individual cells highlight mother/daughter BBs (mothers, red; daughters, green; neither, grey). Starved cells cease BB duplication and contain very few mother/daughter BBs. Scale bar: 15 µm. (E) Quantification of the total number of new BBs at each cell cycle stage. *P<0.05, data is represented as mean±s.d. Significance between groups was determined using an unpaired two-tailed *t*-test. (F) The predicted number of cortical BBs (black bars) based on the observed number of new daughter BBs at each cell cycle stage compared to the observed number of total cortical BBs (red circles and lines; adapted from Fig. 3C). *N*=32 Starved; *N*=127 Stage I, *N*=42 Stage II, *N*=19 Stage III, *N*=11 Stage IV; these values represent the cumulative number of cells imaged from three separate cultures.

At the completion of cell division, a single Stage IV cell undergoes cell division to produce two Stage I cells. Thus, Stage IV cells should have twice the cortical BBs of Stage I cells. However, the average of Stage IV cells harbor only approximately 1.5 fold more cortical BBs than the average of Stage I cells (Stage IV, 592±62 BBs; Stage I, 401±39 BBs; Fig. 3C). This discrepancy may result from new BB assembly during Stage I, which would increase the number of total cortical BBs beyond that predicted by halving the BB content of Stage IV cells. To quantify the frequency of BB assembly during Stage I, we analyzed protein markers for new assembly. The Poc1 BB component incorporates into daughter BBs in an age dependent fashion such that daughter BBs have low Poc1:GFP levels relative to mature BBs (Fig. 4A,B; Fig. S4A,B) (Pearson et al., 2009b). While centrin levels remain relatively constant, Poc1 levels increase as BBs mature (Fig. 4A,B). We tracked new BB assembly events by identifying all BBs whose Poc1 intensity was at least 2-fold less than their posterior neighbor. Consistent with prior reports, nascent BBs appear as a spatial gradient. They are enriched within the medial portion of the cell and exhibit a sharp drop-off at the anterior pole (Fig. 4C) (Kaczanowski, 1978; Nanney, 1975; Pearson et al., 2009a; Perlman, 1973). Using the same 2-fold intensity cutoff, starved cells, which exit the cell cycle and cease BB duplication (Kaczanowski, 1978; Nelsen and Debault, 1978), exhibit fewer than 4% new BBs per cell, which indicates that Poc1:GFP intensity is a marker for new BB assembly (Fig. 4D,E). Stage I cells contain an average of 401 total cortical BBs, and our Poc1 analysis suggests that 70 of these cortical BBs are newly assembled daughter BBs (Fig. 4D,E). By subtracting the 70 daughter BBs from our Stage I cells, we estimate that at the time of cell division *Tetrahymena* harbor approximately 331 cortical BBs before experiencing a period of BB proliferation that raises the average number of Stage I cortical BBs to 401. These results provide evidence of a previously unaccounted population of new BB assembly in Stage I of the *Tetrahymena* cell cycle.

To understand the contribution of new BB assembly to the remainder of the cell cycle, we compared the predicted impact of new assembly events to our observed total BB frequency (Fig. 3C). For example, Stage IV cells have 592 cortical BBs (Fig. 3C), leading to Stage I cells with a predicted 296 cortical BBs (592 BBs divided into two daughter cells=296). Stage I cells should then add 70 daughter BBs (Fig. 4E) during Stage I resulting in an average cortical BB count of 366 BBs, which is 37 BBs fewer (8.6%) than our observed value of 401 cortical BBs. By extending this analysis across the cell cycle, we estimate that our calculations of daughter BB frequency are reduced by 8.6% in Stage I, 7.5% in Stage II, 12.9% in Stage III and 14.6% in Stage IV (Fig. 4F). Thus, the reliability of using Poc1 levels to identify new BBs decreases as the cell cycle progresses. Nonetheless, Poc1 intensity analysis is able to detect a robust period of BB proliferation that begins during Stage I of the cell cycle prior to formation of the oral apparatus.

### The dynamics of *Tetrahymena* BB organization throughout the cell cycle

**Fig. 5.**
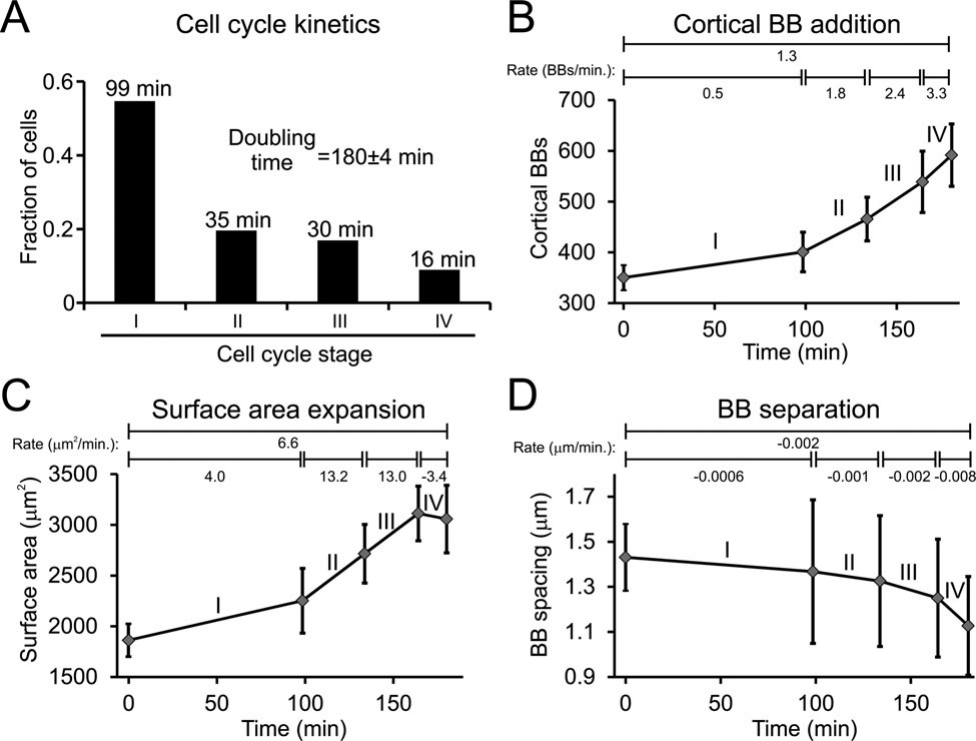
**The dynamics of *Tetrahymena* BB organization.** (A) The fraction of cells at each cell cycle stage in an asynchronous population (doubling time: 180.2 min±3.7 min) reveals the approximate time spent in each stage of the cell cycle. *N*=225 total cells staged from three separate cultures. (B) The average rate of BB addition (BBs/min) increases throughout the cell cycle. (C) The rate of cell surface area expands before plateauing at the end (Stage IV) of the cell cycle. (D) BB spacing decreases at an accelerated rate at the end (Stage IV) of the cell cycle. For (B-D), the 0 min time point is the average parameter value for the quartile of the Stage I population with fewest total cortical BBs. The values above each graph are the rates of change for the entire cell cycle (longest line) or the rate of change for the specific cell cycle stage (short lines). *N*=127 Stage I, *N*=42 Stage II, *N*=19 Stage III, *N*=11 Stage IV; these values represent the cumulative number of cells imaged from three separate cultures. Data is represented as mean±s.d.

The above analyses provide a static snapshot of BB organization at discrete stages of the cell cycle. To gain insight into dynamic changes in BB organization, we calculated the length of time spent in each cell cycle stage by multiplying the relative frequency of cells at each stage by the doubling time of an exponentially growing population. Stage I accounts for more than half of the cell cycle (98/180 min) and cells spend progressively less time at each subsequent stage with Stage IV only lasting 16 min (Fig. 5A). We used this calculated cell cycle timing to investigate the rate of BB addition. To establish a baseline value for the beginning of the cell cycle (0 min) we assumed that the Stage I cells with the fewest total cortical BBs represent the earliest Stage I cells (average cortical BBs in first quartile of Stage I, 350±24 BBs). Importantly, this value is approximately half of the latest Stage IV cells (average cortical BBs in the last quartile of Stage IV, 664±4 BBs), which suggests that the discrepancy between the average number of BBs at each stage (Fig. 3C) results from the coarseness of our staging protocol. In support of this assertion, assuming 350 cortical BBs at the onset of Stage I minimizes the discrepancy between the Poc1 intensity analysis and BB proliferation. Specifically, using the same approach as described for Fig. 4F, our calculations of daughter BB frequency only differ by 4.8% in Stage I, 4.1% in Stage II, 2.9% in Stage III and 5.4% in Stage IV. Although cells add similar numbers of cortical BBs during each cell cycle stage (51 Stage I, 65 Stage II, 73 Stage III, and 53 Stage IV), the rate of BB addition events is dramatically different. During Stage I, cells add BBs at a rate of 0.5 BBs per minute, which sharply increases to 3.3 BBs per minute during Stage IV (Fig. 5B). This increase in the rate of BB addition is mirrored by an accelerated rate of cell surface area expansion through Stage III (Stage I, 4 µm^2^ per minute; Stages II and III, 13 µm^2^ per minute). However, the rate of surface area expansion decreases by Stage IV (−3.4 µm^2^ per minute) indicating that cells contract slightly just prior to cell division. Collectively, these results demonstrate the cell cycle coordination of BB duplication with cell size expansion to ensure that each daughter cell receives a full complement of BBs for proper cellular motility.

### Quantifying the spacing between BBs

Individual cilia within multi-ciliary arrays are spaced at regular intervals, which allows for efficient hydrodynamic flow and coordinated ciliary beating (Elgeti and Gompper, 2013; Marshall and Kintner, 2008). Indeed, spacing defects are commonly found in BB mutants and disease and typically lead to decreased force generation (Park et al., 2008; Pearson et al., 2009b; Rayner et al., 1996). We predict that BB spacing also reflects the frequency of new BB duplication because reduced BB duplication in the absence of cell shrinking leads to increased BB spacing (Bayless et al., 2012). In the relatively flat epithelial sheets of vertebrate cells, 2D projections minimally distort the absolute distance between individual BBs (Mitchell et al., 2007; Werner et al., 2011). However, the problem of cell curvature is more pronounced in ciliates, which has hampered the analysis of BB positioning outside of the medial quadrants (Kaczanowski, 1978; Perlman, 1973).

**Fig. 6.**
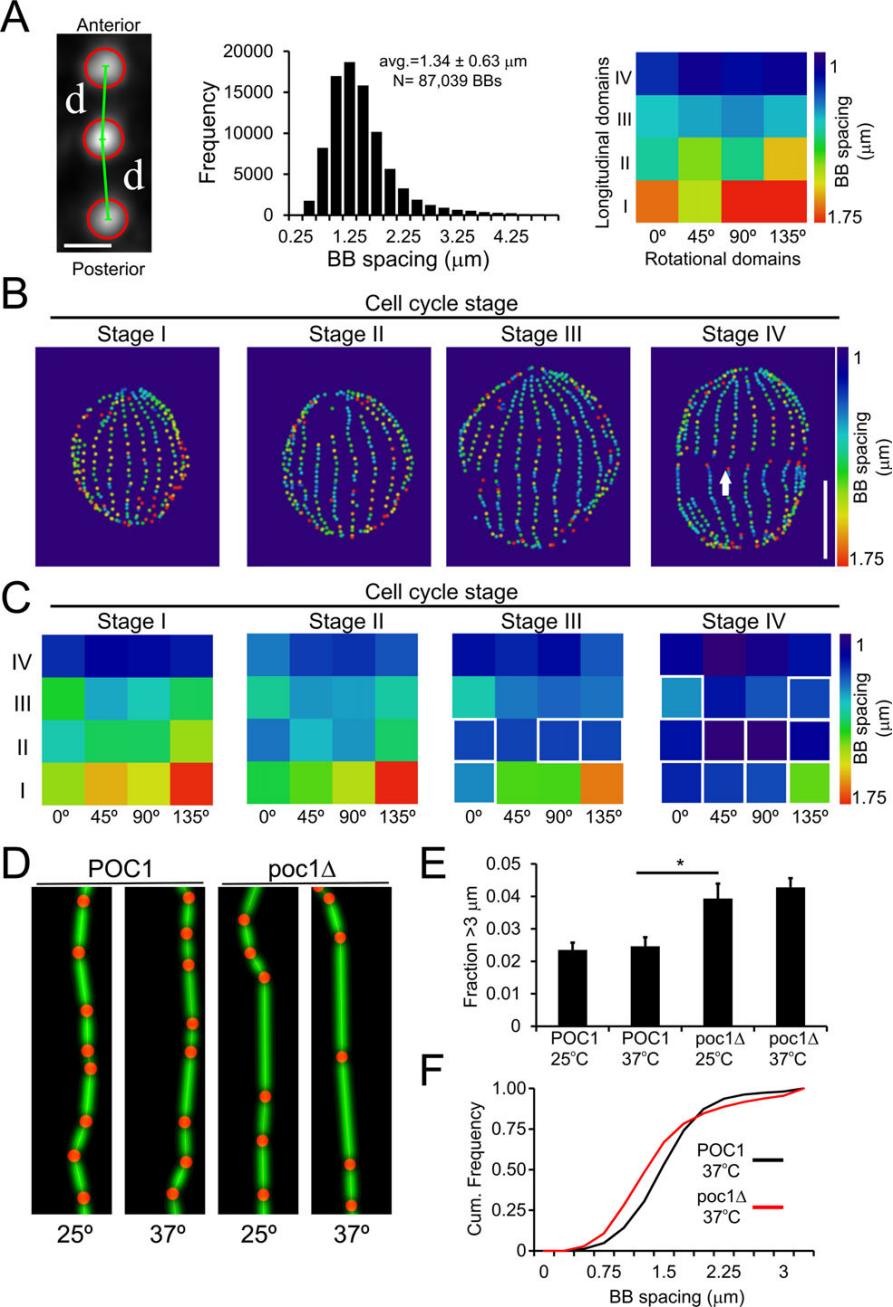
**Automated analysis of BB separation distance identifies spacing differences in cycling wild-type cells and poc1Δ cells.** (A) The image analysis routine tracks the separation distance between BBs and their anterior partner across the entire population (frequency histogram) or at the level of individual spatial domains (heat map; average spacing across the entire data set). Scale bar: 1 µm. *N* represents the total number of basal bodies from 199 cells imaged from 3 separate cultures. (B) Representative maps of the staged cells shown in Fig. 3. The distance between each BB and its anterior partner is color coded. In the Stage IV cell, the arrow highlights the end of ciliary rows at the eventual cleavage plane which are characterized by increased BB spacing. Scale bar: 15 µm. (C) Heat maps of the average BB spacing within each of the 16 spatial domains. White boxes around an individual domain indicate statistical significance (*P*<0.05) versus Stage I cells as determined using a one-way ANOVA and Tukey HSD post-test. *N*=127 Stage I, *N*=42 Stage II, *N*=19 Stage III, *N*=11 Stage IV. (D) Poc1 loss causes a temperature sensitive increase in the frequency of large gaps in BB spacing greater than 3 µm as demonstrated by reconstructions of individual ciliary rows. (E) The frequency of large gaps is nearly doubled in poc1Δ cells. *N*=31 POC1 0 h; *N*=36 POC1 24 h; *N*=34 poc1Δ 0 h; *N*=30 poc1Δ 24 h; these values represent the cumulative number of cells imaged from two separate cultures. *P<0.05, data is represented as mean±s.d. (F) A cumulative frequency plot of BB spacing for POC1 (black) and poc1Δ cells (red) at 37°C demonstrates the increased spacing caused by large gaps (downward shift in the cumulative frequency plot) is compensated for by a corresponding increase in the frequency of closely spaced BBs (leftward shift in the cumulative frequency plot).

To quantify BB spacing across all cellular domains, we measured the separation distance between each BB and its anterior partner (Fig. 6A). Wild-type cells exhibit a gradient of spacing. BBs at the anterior end of the cell (Longitudinal domain IV) display the shortest separation and those at the posterior end of the cell (Longitudinal domain I) exhibit the largest separation (Fig. 6A,C). The average BB spacing (1.34 µm) is in agreement with prior studies that have tracked BB spacing manually (1.3 µm) (Bayless et al., 2012; Pearson et al., 2009b). As cells progress through the cell cycle, BB spacing decreases in the medial and posterior portion of the cell but remains constant at the anterior end of the cell (Fig. 6C; *P*<0.05, white boxes). The BB density per µm^2^ of surface area is relatively constant through Stage III (Fig. 3), but the BB density per linear µm within a ciliary row declines at a rate that increases as cells advance through the cell cycle (Fig. 5D). This indicates that the cell's longitudinal axis does not elongate at the same rate as BB assembly, which causes newly assembled BBs to become packed at a greater density within each ciliary row. Interestingly, the most posterior domain on the dorsal surface exhibited the greatest spacing during each stage of the cell cycle (Fig. 6C), which is predicted to reduce the net power output from that portion of the *Tetrahymena* multi-ciliary array (Elgeti and Gompper, 2013). These results provide a previously undocumented characterization of BB spacing across the 3D landscape of *Tetrahymena* cells.

Loss of Poc1 destabilizes BBs and increases BB spacing in a temperature sensitive manner (Pearson et al., 2009b). In *Tetrahymena*, Poc1's effect on BB spacing has only been analyzed in the medial portion of the cell under cell cycle arrest conditions (Pearson et al., 2009b). To determine how Poc1 globally alters BB spacing during the cell cycle, we analyzed poc1Δ cells at 25°C and after 24 h at 37°C (Fig. 6D). No differences were detected in the average BB spacing in any spatial domain at any temperature. However, analysis of the relative histograms of the entire BB population revealed a shift in the spacing frequency distribution (Fig. S4C). poc1Δ cells had increased BB spacing gaps greater than 3 µm (Fig. 6D,E; Fig. S4D), but the increased gap frequency is balanced by a larger fraction of closely spaced BBs (<1 µm separation; Fig. 6F), which causes the overall average BB spacing in poc1Δ cells to be indistinguishable from wild-type. These results raise the intriguing possibility that *Tetrahymena* harbor an intrinsic BB counting mechanism, whereby large gaps in BB spacing are compensated for by increased BB frequency in other areas of the cell.

### Visualizing BB orientation

**Fig. 7.**
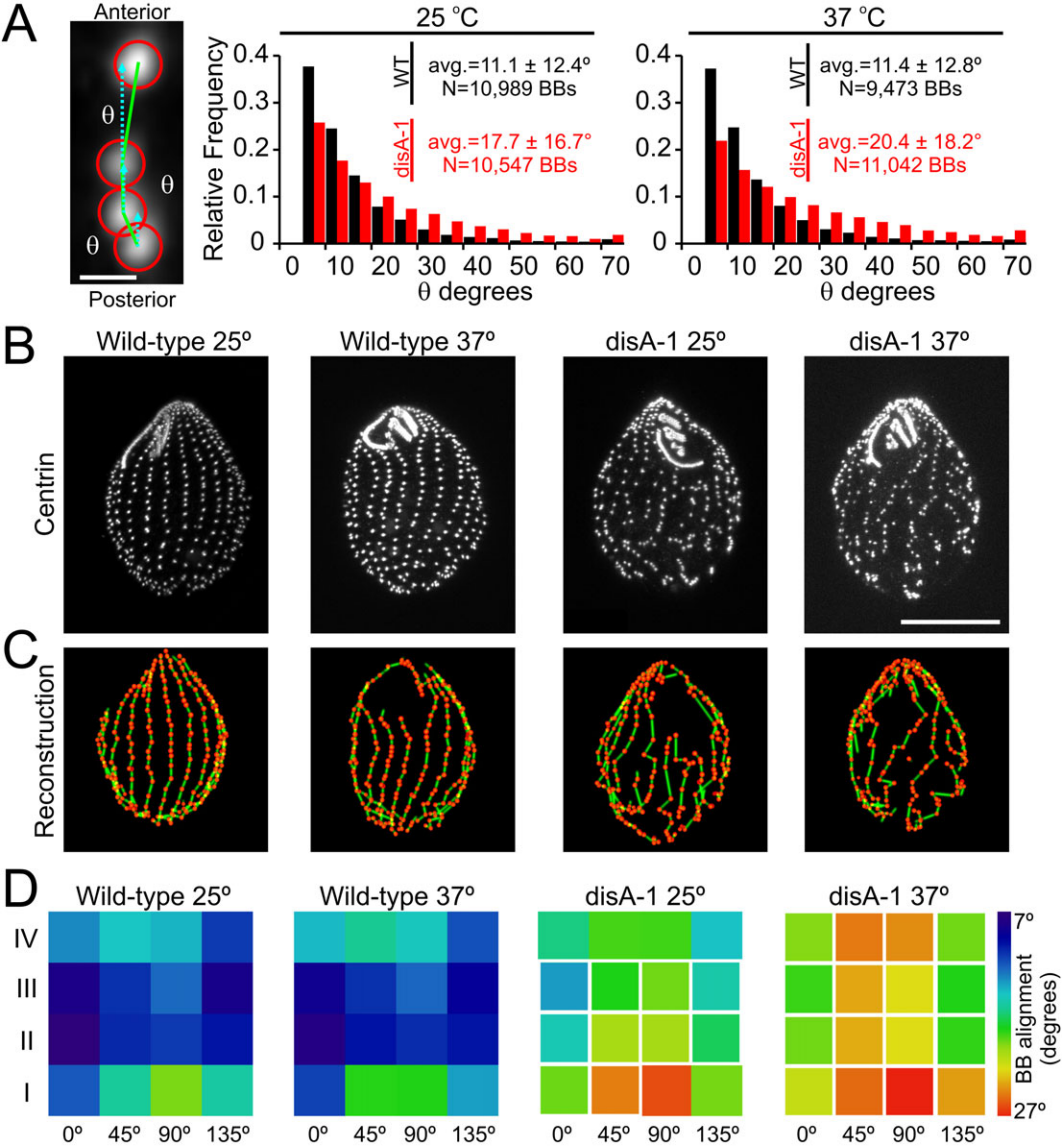
**Automated analysis identifies the domains of BB disorientation.** (A) The image analysis routine tracks the alignment between BBs and their anterior partners by measuring the angular deviation between the anterior BB (green solid line) and a plane passing through the BB of interest and the cellular poles (cyan dotted line). This information can be used to make comparisons across the entire population (frequency histogram) or at the level of individual spatial domains (D, heat map). Scale bar: 1 µm. (B) Cycling wild-type and disA-1 cells cultured at 25°C and 37°C were stained with centrin to mark BBs. Scale bar: 15 µm. (C) Reconstructions of BBs (red spheres) and their anterior partners (green lines). The chaotic connections in disA-1 cells highlights their disorientation, which is more severe at elevated temperatures. (D) The average angular deviation for anterior BBs is displayed as a heat map for each of the 16 spatial domains. At 25°C, increased disorientation occurs in the posterior portion of disA-1 cells. At 37°C the disorientation spreads toward the anterior pole. White boxes indicate statistical significance (*P*<0.05) versus the wild-type cells at 25°C determined using one-way ANOVA and Tukey HSD post-test. *N*=26 cells wild-type at 25°C; *N*=22 cells wild-type at 37°C; *N*=26 cells disA-1 at 25°C; *N*=30 cells disA-1 at 37°C for each condition; these values; these values represent the cumulative number of cells imaged from two separate cultures.

In addition to the total number of BBs and their relative spacing, another critical parameter of BB organization is their alignment or orientation into uniform ciliary rows. In a perfect ciliary row, the anterior partner of a BB is coplanar with the anterior pole, posterior pole and the BB itself (Fig. 7A). Therefore, for a cell with perfect BB orientation the average angular deviation from the plane described above will be 0°, and as the alignment of rows becomes more chaotic the average angular deviation will increase (Fig. 7A). DisAp is a protein component of a BB accessory structure called the kinetodesmal fiber (Galati et al., 2014). The *disA-1* mutation shortens kinetodesmal fibers rendering cells susceptible to temperature sensitive BB disorientation (Galati et al., 2014; Jerka-Dziadosz et al., 1995). The spatial domains where BB orientation in disA-1 cells is most disrupted has not been identified, so we measured orientation in disA-1 cells at 25°C and 37°C (Fig. 7B,C). At 25°C, significant disorientation occurs in the medial and posterior quadrants of disA-1 cells and is most prominent on the cell surfaces lateral to the oral apparatus (Fig. 7D). After 24 h of growth at 37°C, disorientation increases over most of the cell including the anterior pole, which was unaffected at 25°C. These results demonstrate an automated and robust method for measuring the deviation of BB orientation from ciliary rows. Moreover, they show that loss of DisA1p leads to a spatially restricted gradient of BB disorganization. This gradient begins in the posterior portion of the cell and spreads towards the anterior pole in a temperature sensitive fashion.

### Averaging BB protein and structural localization

**Fig. 8.**
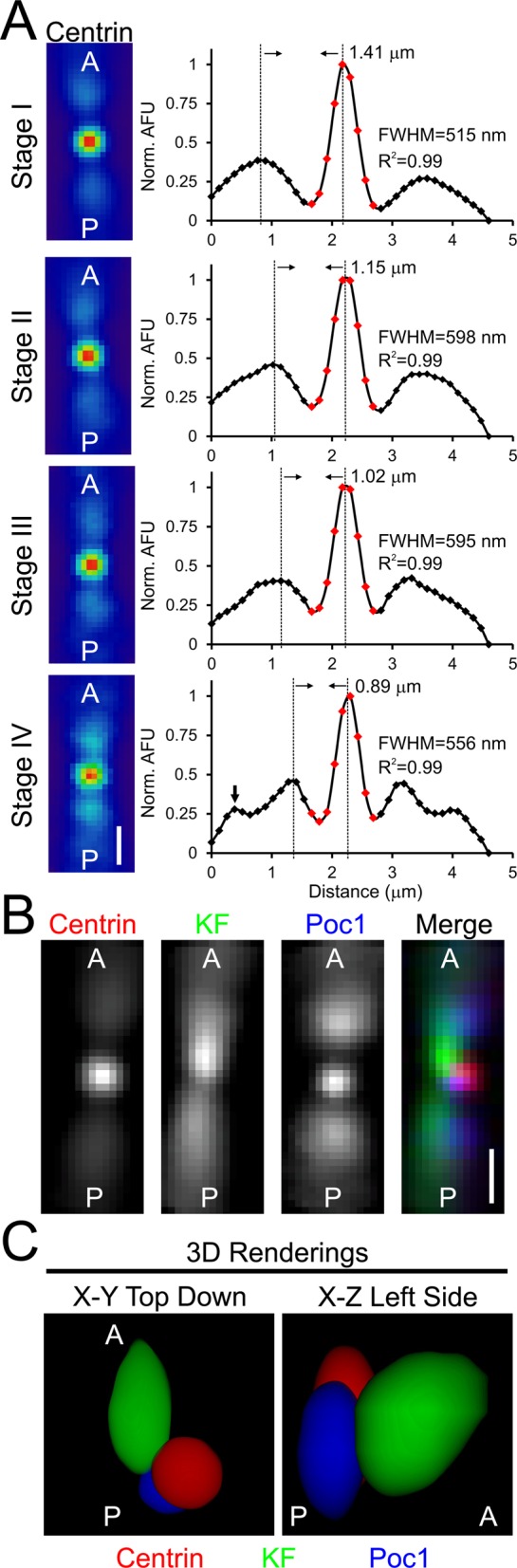
**Reconstruction of individual ciliary units using 3D image averaging.** (A) The average intensity of centrin for each stage of the cell cycle aligned according to the anteroposterior axis of the cell. Averages were generated from BBs in the medial quadrants (II and III). The linescans on the right show the peak fluorescence intensity for the center BB. The smaller symmetric peaks surrounding the center peak correspond to the averaged anterior and posterior partners. As cells progress through the cell cycle the spacing between smaller peaks becomes closer (dashed lines centered through each peak and peak to peak distance are shown) and the number of smaller peaks increases (large black arrow in Stage IV linescan). The nine central data points (red squares) were fit to a Gaussian function and the corresponding R^2^ for the goodness of fit is shown. Scale bar: 1 µm. (B) The average centrin (red), kinetodesmal fiber (KF, green) and Poc1 (blue) signals. The signals were acquired in two different data sets (centrin/KF and centrin/Poc1) allowing centrin to be used as a common fiducial marker. Scale bar: 1 µm. (C) 3D renderings of the average intensity for the three BB components centrin (red), kinetodesmal fibers (green) and Poc1 (blue). The renderings recreate the posterior and proximal shift of the average Poc1 signal relative to centrin as well as the kinetodesmal fiber curvature towards the cell cortex. The renderings were created from cropped data to eliminate the contribution of signal originating from the average anterior and posterior ciliary units.

Fluorescence intensity averaging is a powerful method for localizing structures with sub-resolution accuracy in 3D (Broeken et al., 2015). Such studies helped establish the spatial architectures of macromolecular structures including BBs, centrosomes and kinetochores (Churchman et al., 2005; Gardner et al., 2008; Joglekar et al., 2006, 2009; Pearson et al., 2006; Stemm-Wolf et al., 2013; Wan et al., 2009). The time-intensive step in manual fluorescence averaging is orienting different images along a common axis. Since our image analysis routine determines both the coordinates of each BB as well as cellular polarity, we automated the 3D image averaging of BBs. To demonstrate the accuracy of our approach, we averaged the relative centrin fluorescence intensity in each stage of the cell cycle (Fig. 8A, left). Linescans of the average 2D projections were fit to a Gaussian function and exhibited a strong goodness of fit (R^2^>0.99; Fig. 8A, right). We averaged fluorescence intensity within a cube to capture the fluorescence signal for multiple BBs within a ciliary row. As predicted from our global analysis of BB spacing (Fig. 6), we detected multiple peaks in each average linescan at intervals that match the separation distance of neighboring BBs (Fig. 8A, right). Since the alignment of multiple peaks can only be accomplished if BBs are properly oriented along the anteroposterior axis, these results confirm the accuracy of our automated alignment for high-throughput fluorescence averaging.

Intensity averaging allows multiple proteins to be aligned relative to a fiducial mark, which permits the reconstruction of macromolecular assemblies (Broeken et al., 2015; Stemm-Wolf et al., 2013). To begin reconstructing the *Tetrahymena* BB, we averaged two different data sets that were stained for centrin and either the BB component, Poc1, or the kinetodesmal fiber accessory structure. By orienting each data set to centrin, which is the common fiducial mark between the data sets, a 3D average image was generated. The 3D reconstruction exhibits the expected location of these components based on ultra-structural analysis and immuno-electron microscopy (Fig. 8B,C) (Allen, 1967, 1969; Galati et al., 2014; Pearson et al., 2009b; Stemm-Wolf et al., 2005). Poc1 is centered at the BB base or proximal end whereas centrin localizes anteriorly relative to the cellular geometry and distally at the BB (Paoletti et al., 1996; Stemm-Wolf et al., 2005). The kinetodesmal fiber localizes anterior to both Poc1 and centrin and curves up toward the cell cortex. Thus, automated 3D averaging provides a rapid and accurate means to quantify protein localization within the BB structural architecture.

## DISCUSSION

We developed an automated image processing routine to reconstruct and quantify the 3D organization of *Tetrahymena* cortical BBs. Our analysis routine is robust and accurate; it works on both widefield and confocal images with drastically different intensities and noise profiles and its performance is comparable to a trained human classifier (Fig. S3D,E). We demonstrate the utility of this analysis routine by uncovering important aspects of BB organization in both wild type and mutant cells that are not easily identified without such quantitative analyses. Below we discuss the biological insights gained from our analysis along with its limitations and the potential for improvement.

### BB spacing in wild-type and poc1Δ cells

Determining the spacing between BBs at the anterior and posterior poles of *Tetrahymena* has been difficult due to cellular curvature (Fig. 2G,H) (Kaczanowski, 1978). We found that the greatest average BB separation distance is in the most posterior domain on the dorsal surface and that this cellular trend is maintained throughout the cell cycle (Fig. 3). Cilia spacing plays a critical role in the emergence of metachronal waves (Elgeti and Gompper, 2013). As cilia spacing decreases, the transport velocity of fluid increases and vice versa (Elgeti and Gompper, 2013). Since *Tetrahymena* multi-ciliary arrays beat in a metachronal fashion (Nelsen and Debault, 1978; Wood et al., 2007), we speculate that the greater BB separation distance on the posterior-dorsal surface may be important for *Tetrahymena* motility. Going forward, it will be important to couple detailed morphological characterizations of *Tetrahymena* BB organization with fluid flow experiments to separate the relationship between cellular geometry and force generation.

Defects in BB assembly and/or stability increase the spacing between individual BBs within a ciliary row (Bayless et al., 2012; Pearson et al., 2009b; Ross et al., 2013). These analyses have been performed by manually counting BBs only within spatially restricted portions of ciliary rows, which makes it difficult to understand how BB assembly and stability mutants impact BB spacing at the level of an entire cell. We show that cycling poc1Δ cells display an increased frequency of large BB spacing gaps. However, these gaps do not increase the average spacing of cycling poc1Δ cells because they are offset by an increased frequency in closely spaced BBs, which may indicate a compaction of rows surrounding the large gaps. This observation raises the intriguing possibility that *Tetrahymena* ‘count’ their BBs and increase BB duplication to compensate for large BB gaps. As part of this process, cells may use the spacing of cytoskeletal components as a ruler to gauge BB spacing and initiate the increase in BB duplication (Marshall, 2004). Such a counting mechanism is supported by a compensatory decrease in the number of BBs within a ciliary row as the number of ciliary rows increases (Nanney and Chow, 1974) and the existence of cytoskeletal based molecular rulers that coordinate the length of *Chlamydomonas* flagella with changes in gene expression (Oda et al., 2014). BB counting may parallel the ability of *Tetrahymena* to accurately count chromosomes of the macronucleus in order to maintain 45 copies (Donti et al., 2009; Merriam and Bruns, 1988). Importantly, since non-cycling poc1Δ cells exhibit increased average spacing (Pearson et al., 2009b), we predict that any such BB counting mechanism requires new BB assembly. Since BBs are situated within a cytoskeletal framework that helps orient and maintain BB positioning (Allen, 1967; Werner et al., 2011), it will be interesting to test whether BBs separated by large gaps are more susceptible to force-dependent disorganization or disassembly (Bayless et al., 2012; Galati et al., 2014).

### Balancing BB assembly and organization

Poc1 incorporates into BBs in an age-dependent fashion, which causes daughter BBs to have reduced Poc1:GFP fluorescence intensity relative to their posterior partners (Pearson et al., 2009b). We took advantage of this incorporation profile to automatically identify daughter BBs in staged cycling cells. This task was previously achieved by manually counting BBs that lack cilia, which presumably represent newly assembled daughter BBs (Nanney, 1975; Perlman, 1973). Consistent with prior observations (Kaczanowski, 1978; Pearson et al., 2009a), the majority of daughter BBs (dim Poc1:GFP) were present in the medial portion of the cell and found during each stage of cell division (Fig. 4, Fig. S4A,B). Moreover, similar to (Nanney, 1971), our BB counts at the latest stage of the cell cycle (Stage IV) are fewer than would be expected based on BB counts from the earliest stage of the cell cycle (Stage I). Two models have been put forth to explain this discrepancy (Kaczanowski, 1978; Nanney, 1975). First, BB assembly begins early in the cell cycle, prior to oral apparatus development, which increases the average cortical BBs in Stage I beyond the number of BBs found immediately following division (Nanney, 1975). Second, BB assembly continues right up until the time of cell division, and since it is difficult to capture cells at this late phase, the average cortical BBs in Stage IV are an underrepresentation of the BBs present immediately prior to division. While our analysis does not permit us to exclude the contribution of BB assembly just before cell division, we identify new assembly events in Stage I cells. This suggests the first model where substantial BB assembly occurs during early stages of the cell cycle.

Prior analyses found that the fraction of newly assembled BBs increases through the cell cycle (Kaczanowski, 1978; Nanney, 1975; Perlman, 1973). In contrast, our analysis revealed that the fraction of newly assembled BBs decreases in the later stages of the cell cycle, which is unlikely based on our observed increase in total cortical BBs through cell division (Fig. 3). Two factors may contribute to this difference. First, daughter BBs accumulate in linear clusters that increase in size as cells mature (Kaczanowski, 1978). Since we defined newly assembled BBs as those with at least a 2-fold intensity decrease relative to their posterior partner, our analysis underestimates low Poc1:GFP intensity BBs that reside in linear clusters. This is because only the first BB of a linear cluster of dim Poc1 BBs will be identified as a newly assembled daughter BB. Future versions of the analysis routine will assign each BB a position within an individual ciliary row to enhance our detection of linear clusters of newly assembled daughter BBs. Second, it has been suggested that the lag time between BB assembly and ciliation may be as long as one full cell cycle (Nanney, 1975), which would lead to a steady accumulation of non-ciliated BBs throughout a single cell cycle. Our data is consistent with the notion that BBs recruit a mature level of Poc1 in less than one cell cycle. Rather than providing a cumulative count of all newly assembled BBs, our analysis provides a snapshot of the positioning of the most nascent daughter BBs. In the future, the accuracy of intensity based identification of BB assembly can be enhanced by tracking multiple markers of BB maturity or by identifying maturity markers that accumulate with slower kinetics. Regardless, our analysis of Poc1 intensity provides firm evidence for a substantial population of newly assembled BBs at the early stages of the cell cycle.

### Measuring BB orientation

The alignment of individual BBs with cellular polarity is essential for generating strong hydrodynamic force (Elgeti and Gompper, 2013; Marshall and Kintner, 2008). The *Tetrahymena disA-1* mutation disorients BBs and their alignment by disrupting the length of the kinetodesmal fiber (Galati et al., 2014; Jerka-Dziadosz et al., 1995). However, the spatial domains of disorientation were not quantified. BB disorientation in disA-1 cells grown at 25°C is most severe in the medial and posterior regions of the cell (Fig. 7), while cells shifted to 37°C exhibit increased BB disorientation within the same medial and posterior regions, but the disorientation also spread to the anterior domains of the cell. This indicates that the anterior tip of the cell harbors mechanisms that allow it to resist disorienting stimuli, even in the face of shortened kinetodesmal fibers. In addition to an anterior-posterior gradient of disorientation, we also identified a rotational gradient with the disorganization being most severe on the sides of the cell, which may reflect that these side domains experience greater force leading to increased disorientation. Going forward, we envision that additional high-throughput analyses of BB organization using similar computational routines will increase our understanding of how the geometry of multi-ciliary arrays informs fluid flow and cellular motility in both *Tetrahymena* and multi-ciliated vertebrate cells.

## MATERIALS AND METHODS

### Tetrahymena culture

*T. thermophila* cells were grown in 2% SPP media (2% proteose peptone, 0.2% glucose, 0.1% yeast extract, and 0.003% Fe-EDTA) at the indicated temperatures. Cells were analyzed at mid-log phase (density between 1×10^5^ and 4×10^5^ cells/ml) as determined using a Coulter Counter Z1 (Beckman Coulter). For the starvation experiment, cells were arrested in the G1 phase of the cell cycle by culturing in 10 mM Tris-HCl, pH 7.4, for 18-24 h. The wild-type strain for the cell cycle analyses was B2086 transformed with the Poc1:GFP plasmid described in (Pearson et al., 2009b). The poc1Δ strain was described in Pearson et al. (2009b). The wild-type strain (B1868) and disA-1 strain (IA217) were described in Galati et al. (2014) and Jerka-Dziadosz et al. (1995).

### Immunofluorescence microscopy

Immunofluorescence was performed as previously described (Galati et al., 2014). Briefly, 1-3×10^5^ cells were pelleted at 1500 ***g*** and fixed for 20-30 min with 70% ethanol+0.2% Triton X-100. Cells were washed with 10 mM Tris-buffered saline and blocked overnight at 4°C in 1% BSA in 10 mM TBS. Cells were immunostained by incubating overnight at 4°C in primary antibody [mouse anti-KF (5D8), 1:400, a gift from J. Frankel, University of Iowa, (Williams et al., 1995); rabbit anti-centrin, 1:2000, a gift from A. Stemm-Wolf and M. Winey, University of Colorado Boulder, (Stemm-Wolf et al., 2005); chicken anti-GFP; Abcam; catalog number ab13970]. Secondary antibody incubations were performed for 1 h at room temperature (goat anti-mouse Alexa Fluor 594, 1:2000; goat anti-chicken Alexa Fluor 488, 1:2000; goat anti-rabbit Alexa Fluor 647, 1:2000; Invitrogen). Cells were mounted in Citifluor mounting media (Citifluor) using #1.5 coverslips and sealed with nail polish.

Microscopy was performed as previously described (Bayless et al., 2012; Galati et al., 2014). Briefly, for the widefield images in Fig. S2C,D, an inverted microscope (Ti Eclipse; Nikon) with a 100× Plan-Apochromat (NA 1.4) objective lens (Nikon) was used. Images were captured with a scientific CMOS (sCMOS) camera (Xyla 4.2, Andor Technology). For all other experiments, confocal microscopy was performed using an inverted microscope (Ti Eclipse) with a 100× Plan-Apochromat (NA 1.43) objective lens (Nikon) and a Swept Field confocal scan head (Prairie Technologies). Confocal images were acquired in slit mode with a slit size of 35 µm and a *z*-step size of 300 nm, and detected with a charge-coupled device (CCD) camera (Clara, Andor Technology). Images were acquired with Elements software (Nikon) and all fixed cells were imaged at room temperature.

### Digital image quantification

We developed an image analysis routine to quantify the BB content and cell shape from digital images of *Tetrahymena thermophila* cells immunostained with BB markers (centrin and Poc1:GFP). The routine exploits stereotypical aspects of *Tetrahymena* organization to: (1) extract the location of a *Tetrahymena* cell within a 3D image stack, (2) identify the absolute location of BBs along with their relative position within ciliary rows, (3) calculate the volume and surface area of individual cells and (4) orient these features relative to the cell's polarity. The routine was implemented with the ImageJ/FIJI macro language and the code is available along with a detailed set of instructions for how to use the code and sample images (http://www.thepearsonlab.com/publications.html). Below is a description of the routine features.

#### Identify individual *Tetrahymena* cells

Large radius blurring followed by histogram thresholding extracts the 2D outline of complete *Tetrahymena* cells from a field of view contaminated with background, debris and partial *Tetrahymena* cells. Briefly, a maximum intensity projection (MIP) is created and the mean pixel intensity is subtracted from the MIP. The background subtracted MIP is convolved with a large radius Gaussian kernel (1 µm radius) to homogenize individual BB fluorescence into the large cellular scale features. Large features are separated by convolving with a small radius Laplacian of Gaussian kernel (0.12 µm radius) and further smoothed with another large 1 µm Gaussian kernel. The resulting MIP is thresholded using the Triangle method (Zack et al., 1977) and segmented objects are filtered based on their shape. Objects greater than 2000 µm^2^ in 2D area or that have a maximum Feret diameter greater than 60 µm or that have a circularity of less than 0.85 are rejected. The largest remaining object that is retained is the cell of interest.

#### Identifying *Tetrahymena* BBs

Next, individual BBs are identified using low radius Gaussian filtering combined with 3D local maxima finding. The image stack is cropped with the cell mask generated above (enlarged by 5 µm to account for irregularities in the mask) and convolved with a low radius Gaussian kernel (0.12 µm radius; ∼full-width half maximum for a 240 nm diameter BB). Local maxima are then identified using a 0.25 µm *x*-*y* search radius and a 0.6 µm *z* search radius, which correspond to the approximate dimensions of an individual BB. Since the image stack is not background subtracted, the local maxima search yields an oversampling of intensity peaks within the image, such that the majority of peaks correspond to a mixture of camera noise, fluorescence staining noise and cellular debris. An adaptive thresholding approach is used to separate noise from peaks corresponding to actual BB maxima. To calculate the adaptive threshold, the average intensity and standard deviation for all peaks is calculated within a rolling average with a z range of 1.5 µm (Fig. S1). For a given plane, the intensity threshold is defined as ((6−(I_cv_×6))×I_s.d__._)+I_avg_, where I is maxima intensity, cv is coefficient of variation, s.d. is standard deviation and avg is average. This thresholding approach allows the reliable separation of noise maxima from BB maxima so long as the BB's maximum intensity is greater than the noise's average maximum intensity (Fig. S1). Moreover, since the threshold is adaptive in *z*, BB maxima within deep *z* planes are identified despite the significant intensity attenuation that results from light scattering at image planes far from the coverslip (Hell et al., 1993; Waters, 2009).

Our thresholding approach robustly separates noisy maxima from BBs but it does not discriminate cortical BBs from high intensity internalized BBs or oral apparatus BBs. To remove BB maxima within the interior of the cell, a 3D convex hull is generated from the BB maxima and all maxima that are greater than 2.25 µm from the surface of the convex hull are identified and deleted by measuring the intensity of 3D Euclidean distance map of the 3D convex hull (3D ImageJ plugins; Ollion et al., 2013). Since the oral apparatus contains hundreds of BBs that are spaced at distances that approach the diffraction limit in confocal microscopy the oral apparatus appears as a collection of peaks within a homogenous, high intensity background. This is in contrast to cortical BBs that appear as Gaussian peaks that decay to cellular background. To distinguish between these two BB classes, a 1 µm^2^ box is centered over the BB maxima and the skew and kurtosis of the intensity histogram is calculated from a background subtracted image (Fig. S2A,B). Cortical BBs have a positive skew and kurtosis, while oral apparatus BBs have a negative skew and kurtosis due to their homogenous background. BBs are excluded if the sum of their skew and kurtosis is less than 1.

#### Analysis of BB organization and distribution

The output of the above image analysis routine is a list of points that correspond to the location of cortical BBs within a single *Tetrahymena* cell. This list of points is subjected to various analyses in order to extract cellular parameters. To identify the poles of the cell, the maximum distance between each BB and every other BB is determined and a list of the 10 greatest distances is created. These distances correspond to the 10 most anterior BBs and the 10 most posterior BBs and the centroid of each of these clusters of BBs is taken to be the anterior and posterior pole respectively. The surface area and volume of the cell is calculated based on the 3D convex hull of the cortical BB point cloud. BBs are assigned a position within a cortical row or kinety by using a metric that minimizes the distance between each BB and its partner while also minimizing the distance between its partner and a plane comprising the 3D coordinates of the BB maxima, the anterior pole and the posterior pole. This metric is calculated in both the forward and reverse directions and BBs are assigned a partner only if the forward and reverse directions identify the same reciprocal BB pair. Once a BB is assigned a partner, it is disallowed from receiving additional connections. This process is repeated until all connections are made. At the end of the connection process, the only unconnected BBs that remain are those that will never yield reciprocal connections. These BBs are connected to the closest unconnected BB only if the connection is less than 5 µm. If the connection is greater than 5 µm the BB is not connected. This procedure connects the vast majority of BBs with partners in both the posterior and anterior direction, which allows BB separation distances and BB intensity ratios between BBs and their neighbors to be calculated. Moreover, the angular deviation of the anterior BB partner from the plane containing the BB and the anterior and posterior axis provides a measure of the organization and orientation of a BB and the anterior partner. Specifically, in a perfect ciliary row each anterior BB is within the plane defined by BB_xyz_, AntPole_xyz_ and PostPole_xyz_ and thus will have an angular deviation of 0° from this plane. BBs are globally classified according to their Euclidean distance from the anterior pole of the cell, which facilitates their grouping into spatial domains along the anterior-posterior axis. Similarly, BBs are classified according to their angular rotation from the oral apparatus which is defined as the centroid of the homogeneous peaks described above. Collectively, this computational analysis routine provides a comprehensive analysis of all cortical BBs and their location relative to one another as well as their location relative to the cell's global polarity.

### Statistical analysis and data representation

Data was analyzed using a combination of ImageJ/FIJI (NIH), Microsoft Excel and GraphPad Prism. Multiple comparisons were made using a one-way ANOVA with a Tukey HSD post-test with a significance cutoff of *P*<0.05 using GraphPad Prism. Single comparisons were made using an unpaired two-tailed *t*-test using Microsoft Excel with a significance cutoff of *P*<0.05. For all statistical tests, the number of cells was used as *N*. Figures were prepared using a combination of ImageJ/FIJI, Microsoft Excel and Corel Draw. 3D reconstructions were generated using the ImageJ 3D viewer (Schmid et al., 2010). Unless noted, all error bars represent one standard deviation from the mean. Sample sizes were determined from preliminary experiments to find the minimum number of cells needed to show a statistically significant difference in the total number of basal bodies between Stage I and Stage IV cells. Cells were excluded from the analysis if they were severely disfigured from the staining/mounting procedure or if they had high cytoplasmic centrin levels.
